# Interaction of sexual dimorphism and gene dosage imbalance in skeletal deficits associated with Down syndrome

**DOI:** 10.1016/j.bone.2020.115367

**Published:** 2020-07

**Authors:** Jared R. Thomas, Jonathan LaCombe, Rachel Long, Eva Lana-Elola, Sheona Watson-Scales, Joseph M. Wallace, Elizabeth M.C. Fisher, Victor L.J. Tybulewicz, Randall J. Roper

**Affiliations:** a Department of Biology, Indiana University-Purdue University, Indianapolis, IN, USA; b The Francis Crick Institute, London, UK; c Department of Biomedical Engineering, Indiana University-Purdue University, Indianapolis, IN, USA; d Institute of Neurology, University College London, UK; e Department of Immunology & Inflammation, Imperial College London, London W12 0NN, UK

**Keywords:** Trisomy 21, Skeletal abnormalities, Genetic animal models, Developmental modeling, Osteoporosis, Sexual dimorphism

## Abstract

All individuals with Down syndrome (DS), which results from trisomy of human chromosome 21 (Ts21), present with skeletal abnormalities typified by craniofacial features, short stature and low bone mineral density (BMD). Differences in skeletal deficits between males and females with DS suggest a sexual dimorphism in how trisomy affects bone. Dp1Tyb mice contain three copies of all of the genes on mouse chromosome 16 that are homologous to human chromosome 21, males and females are fertile, and therefore are an excellent model to test the hypothesis that gene dosage influences the sexual dimorphism of bone abnormalities in DS. Dp1Tyb as compared to control littermate mice at time points associated with bone accrual (6 weeks) and skeletal maturity (16 weeks) showed deficits in BMD and trabecular architecture that occur largely through interactions between sex and genotype and resulted in lower percent bone volume in all female and Dp1Tyb male mice. Cortical bone in Dp1Tyb as compared to control mice exhibited different changes over time influenced by sex × genotype interactions including reduced cortical area in both male and female Dp1Tyb mice. Mechanical testing analyses suggested deficits in whole bone properties such as bone mass and geometry, but improved material properties in female and Dp1Tyb mice. Sexual dimorphisms and the influence of trisomic gene dosage differentially altered cellular properties of male and female Dp1Tyb bone. These data establish sex, gene dosage, skeletal site and age as important factors in skeletal development of DS model mice, paving the way for identification of the causal dosage-sensitive genes. Skeletal differences in developing male and female Dp1Tyb DS model mice replicated differences in less-studied adolescents with DS and established a foundation to understand the etiology of trisomic bone deficits.

## Highlights

• Male and female Down syndrome (DS) mice have distinct differences in skeletal deficits.
• Effects of trisomy on bone properties are age and sex dependent.
• Different cellular mechanisms may cause bone deficits in male and female DS mice.
• Findings of skeletal deficits in DS mice correlate to observations in people with DS.

## 1 Introduction

Trisomy 21 (Ts21) affects ~1/800 live births and results in distinctive craniofacial features and skeletal deficits, including short stature in all individuals with Down syndrome (DS). Alterations in skeletal features associated with DS begin prenatally and continue postnatally; adolescents exhibit low bone accrual during puberty, and adults have documented bone loss [[1], [2], [3]]. Fetuses with Ts21 are often characterized ultrasonically with a short femur and humerus, but these soft markers are not diagnostic [[4], [5], [6], [7]]. At birth, infants with DS are shorter than typically developing individuals [8,9] and on average, this reduced height continues throughout life [2]. The short stature caused by Ts21 is related to a delay in the development of the secondary centers of ossification and final peak height reached around the age of 15 [1,10]. Peak bone mass is reached ~5–10 years earlier than normal and is lower in individuals with DS as compared to the general population [11]. This predisposition to weaker and shorter bones in people with Ts21, through attenuated bone accrual or altered organization, may be exacerbated due to hypotonia, hormonal and nutritional deficits, growth retardation, low muscle strength and reduced physical activity [[12], [13], [14]].

Therapeutic advancements have brought average expected DS lifespan up to ~60 years [[15], [16], [17]], but individuals with DS are thus increasingly at risk for low bone mineral density (BMD) and fractures. Skeletal deficiencies and lack of bone mass accrual during development predispose people with Ts21 to fragility fractures, and at later ages, osteoporosis [2,13,18,19]. All people with DS are at risk for osteoporosis and osteopenia [10,20,21]; and a high percentage of individuals with DS have suffered fractures [22,23]. Though the risk for osteoporosis is higher in adults with intellectual disabilities, the overall bone density screening rates are lower in these same individuals [24]. Individuals with DS exhibit many hallmarks associated with osteoporosis including a lower BMD, altered geometry, and reduced overall strength. These skeletal deficits found in DS are not only age- and sex-dependent, like normally developing individuals, but are also influenced by having three copies of the genes on human chromosome 21 (Hsa21). Because of the known skeletal abnormalities in all people with DS and the increasing age in the same population, there is a critical need to address the etiology of these skeletal abnormalities.

Some differences in bone properties between males and females with Ts21 have been observed, but low sample sizes, variety of analysis methods, wide array of ages, different skeletal measurement sites makes comparisons between studies difficult [23]. Thus the etiology of DS sexual dimorphism remains unclear. Adolescent females with DS are hypothesized to acquire BMD at a lower rate compared to adolescent males with DS [2,3]. BMD is decreased in the lumbar spine of 12–16 year old males and females (analyzed together) with DS compared to age matched adolescents [25]. Twenty adolescents with DS (males and females analyzed together) compared to 20 males and females without DS showed a lower BMD in lumbar spine, hip, and whole body [26]. However, no differences were seen in femur BMD between males and females with Ts21 in a study of 12 male and 14 female adolescents with DS [27]. When the lumbar vertebrate of males and females (average age ~25 years) with and without DS were analyzed, males with DS as compared to controls had 25% lower BMD and females with DS had 14% lower BMD [1]. Males with Ts21 have been reported to have greater BMD and bone area in the upper and lower limbs than females with DS [2]. Additionally, osteopenia has been reported to occur earlier in males with DS [28], and unlike the most common forms of osteoporosis where 80% of the affected individuals are women, osteoporosis affects both men and women with Ts21. Differences in calculations of BMD using T- or *Z*-scores to determine a low bone mass or osteoporosis add to the variation and bias in individuals with DS [23].

Recent publications detail skeletal deficiencies across life stages in males and females with DS in larger sample sizes and compared to normal developing populations. When femoral neck and lumbar spine BMD were measured in adult males and females with Ts21 ages 20–69, both males and females with DS as compared to reference samples showed lower lumbar spine and femoral neck BMD throughout their lives [14,29]. Males with DS had reduced femoral neck BMD beginning in their 20s but females with DS did not show reduced femoral neck BMD until their 40s. When 128 adults with DS were compared to age-matched adults without DS and other intellectual disabilities, lumbar spine BMD in men was relatively stable in early to mid-adulthood, and bone mineral accrual was later in women than men with DS [30]. Women with DS also saw a rapid loss of bone mass after age 40. Both men and women with DS had higher rates of osteopenia and osteoporosis than a reference population. Taken together, these data indicate different skeletal deficits seen in males and females with DS, but they are complex in their nature, and not well defined in relation to age, skeletal site, and fracture risk.

Mouse models recapitulate the genetic (trisomy) and phenotypic (DS-associated traits) features attributed to Ts21 [[31], [32], [33]] and are utilized to understand skeletal phenotypes associated with DS. The Ts(17^16^)65Dn mouse (Ts65Dn), the most widely used and well-characterized mouse model of DS, has a small trisomic chromosome that contains ~104 genes and 13 Mb that correspond to Hsa21 [34,35]. Adult Ts65Dn male mice show structural and biomechanical skeletal deficits [[36], [37], [38]]. A significant increase in osteoclast surface and osteoclast number was observed in 6 week old Ts65Dn compared to wild-type male mice [39]. Overall, studies in mice and humans imply that trisomy causes developmental bone abnormalities, compromised bone strength, and early onset osteoporosis due to a combination of altered developmental, homeostatic and resorptive bone mechanisms [36,37,39].

Though skeletal differences between normal and trisomic bone have been established using DS mouse models, little has been done to examine the effect of sex on trisomic skeletal structure, mechanics, and cellular composition, mostly because female Ts65Dn mice are reserved for colony maintenance. Additionally, Ts65Dn mice contain three copies of ~35 protein coding genes from mouse chromosome 17 (Mmu17) that are orthologous to Hsa6 [32,40,41] and the contribution of these genes to skeletal phenotypes is unknown. No differences in BMD were seen at 3 or 16 weeks in male or female Ts1Rhr mice with trisomy for 33 orthologous Hsa21 genes (4.2 Mb), likely due to the ages sampled and the limitations of the methodology used to characterize the skeletal defects in these animals [42]. C57BL/6J.129P2-Dp(16Lipi-Zbtb21)1TybEmcf/Nimr (Dp1Tyb) mice have a duplication on Mmu16 *Lipi* to *Zbtb21* spanning 23 Mb and 148 coding genes and contain three copies of all of the genes on Mmu16 that are orthologous to Hsa21 [43]. Both male and female Dp1Tyb mice are fertile which facilitates examination of skeletal features of both sexes in this mouse model. We hypothesized that Dp1Tyb male mice would have similar bone phenotypes as Ts65Dn male mice and that female mice would have a distinct bone phenotype as compared to male DS mice, which will establish a sexually dimorphic phenotype in the appendicular skeleton of a novel DS mouse model. Additionally, we hypothesized bone abnormalities would be differentially affected during longitudinal growth periods of bone accrual and the onset of skeletal maturity.

## 2 Materials and methods

### 2.1 Animals

C57BL/6J.129P2-Dp(16Lipi-Zbtb21)1TybEmcf/Nimr (Dp1Tyb) mice [43] were bred at the Medical Research Council (MRC) Harwell Institute, UK by crossing to C57BL/6J mice. Genotyping was undertaken using custom probes (Transnetyx). Female mice were nulliparous. Femurs were isolated from Dp1Tyb and littermate wild-type mice at 6 and 16 weeks of age. Right femurs were stored in phosphate buffered saline (PBS)-soaked gauze, shipped on dry ice and stored at −80 °C to be used for microcomputed tomography (μCT) analysis and mechanical testing. Left femurs were stored in 70% ethanol and analyzed for dynamic and static histomorphometry. From our previous skeletal analyses, we estimated that we would need ten mice of each genotype to quantify both trabecular and cortical bone parameters (α = 0.05, 1- β = 0.80 in power analyses). Femurs from 6-week-old male (n = 10 control, n = 10 Dp1Tyb) and female (n = 12 control, n = 11 Dp1Tyb); and 16-week-old male (n = 13 control, n = 12 Dp1Tyb) and female (n = 19 control, n = 22 Dp1Tyb) were used. All assessment of femurs was done with investigators blind to sample genotype. Histomorphometry was only completed on 16-week-old male and female mice. All regulated procedures were carried out with approval from a Local Ethical Review Panel and under authority of a Project License granted by the UK Home Office, and in accordance with EU Directive 2010/63/EU.

### 2.2 Preliminary skeletal analysis

DEXA was performed on Dp1Tyb and control mice at 14 weeks as described in https://www.mousephenotype.org/impress/protocol/90/7. Each X-ray image included a standard measurement of 20 mm. Femur (measured from proximal epiphysis to distal epiphysis) and rostrocaudal (tip of the soft tissue of the nose to base of tail) lengths were measured via ImageJ. The femur was analyzed due to its prevalence in the Jackson Laboratory Phenome database and previous work in DS mouse models.

### 2.3 Microcomputed tomography (μCT) analysis

Femurs were thawed to room temperature and scanned using high-resolution μCT system, SkyScan 1172 microCT (SkyScan, Kontich, Belgium) using the following parameters: voltage 60 kV, 12 μM nominal voxel size, binning mode 2 k and filter Al 0.5 mm. Calibrations were performed daily using two cylindrical hydroxyapatite phantoms (0.25 and 0.75 g/cm^3^ calcium hydroxyapatite). Femurs were rewrapped in PBS-soaked gauze and stored at −80 °C until mechanical testing. Reconstruction analysis was performed using NRecon and CTan software (Skyscan, Bruker microCT, Belgium). Trabecular and cortical analyses were accomplished using previously published protocol [44,45].

Trabecular analysis was performed on the distal metaphysis with a region of interest (ROI) defined as 10% of the total bone length, approximately 1 mm proximal to the distal growth plate and then extending proximally. The ROI was auto-segmented using a custom Matlab (MathWorks, Inc. Natick, MA) code [45]. Measurements of trabecular architecture (bone volume fraction [BV/TV], trabecular thickness, number, and separation), bone mineral density (BMD), and tissue mineral density (TMD) were calculated using CTAn.

Cortical analysis was performed at a standard site 60% of the total bone length away from the distal growth plate. Seven transverse slices were generated from the standard site and cortical geometric properties were obtained from using a custom Matlab code.

### 2.4 Mechanical testing

The mechanical properties of the femur were determined by 3-point bending using a mechanical testing machine (TA ElectroForce 3200; Eden Prairie, MN, USA). The femurs were thawed to room temperature and tested in the anterior-posterior direction with the posterior surface in compression (7 mm support span). The load was applied to the midpoint of each bone. The femur was preloaded using 0.5 N to establish contact with the bone and then monotonically tested to failure at a displacement rate of 0.025 mm/s while fully hydrated. Load and deflection were recorded, from which structural strength (yield and ultimate), stiffness (slope of the linear portion of the force versus displacement curve), and deformation (yield deformation, postyield deformation and total deformation) were determined [46,47]. Distance from the proximal end of the femur to the location of the fracture initiation was measured using calipers. Seven transverse slices were obtained from μCT at the location of fracture and calculated geometric properties (bending moment of inertia and distance from the centroid to the tensile surface of the bone). Along with the deflection data, the moment of inertia and distance from the centroid to the tensile surface of the bone in tension derived were used to map load-displacement into stress vs. strain curves using standard equations derived from Euler-Bernoulli beam theory to estimate tissue level properties [46]. The mechanical strength, stiffness, and work/toughness were determined from the force vs. displacement and stress vs. strain curve.

$$\mathrm{Stress}=\sigma =\frac{\mathrm{Fac}}{2I_{\mathrm{AP}}}\left(\mathrm{MPa}\right)$$

$$\mathrm{Microstrain}=\mu \varepsilon =\frac{6\mathrm{cd}}{a\left(3L-4a\right)}\times 10^{6}$$

In these equations, F is the force, d is the displacement, a is the distance from the support to the inner loading point (4 mm), and L is the span between the outer supports (7 mm). The yield point was calculated using the 0.2% offset method based on the stress-strain curve. The modulus of elasticity was calculated as the slope of the linear portion of the stress-strain curve.

### 2.5 Histomorphometric analyses

Male and female mice were injected intraperitoneally with 0.6% Calcein green diluted in PBS at 15 weeks of age, and injected again 4 days later. Three days after the second injection of Calcein green, the mice were euthanized and weighed (16 weeks of age). Femurs were removed and placed in 70% ethanol and stored at room temperature until ready for use. The femur was separated at the midshaft and the proximal and distal femurs were processed, cut, and sectioned as described [36]. Briefly, femurs were dehydrated in graded levels of ethanol, cleared in xylene and embedded in methyl methacrylate. For dynamic histology, the midshaft of the femur was sectioned into 500 μm transverse sections and ground to 40 μm and then mounted using Eukitt to enhance viewing of fluorescent label. One section was read using a D-FL Epi-Fluorescence attachment on a Nikon Eclipse 80i DIC microscope, and images were taken using a Nikon DS-Fi1 digital sight camera. Mineralizing surface/bone surface (MS/BS) was assessed by measuring the double label surface (dL.S), single label surface (sL.S), and total bone surface (BS) using BioQuant software (R & M Biometrics, Nashville, TN; MS/BS = (dL.S. + 0.5 ∗ sL.S)/BS). Mineral apposition rate (MAR) was determined by measuring the average interlabel width between two fluorochrome labels using the Bioquant software, divided by the number of days between label administration. MS/BS and MAR were used to quantify bone formation rate (BFR = MS/BS ∗ MAR ∗ 365; μm^3^/μm^2^/year). Based on the measurements obtained from dynamic labeling, some mice had to be removed from analysis: for the periosteal surface 16-week-old male (n = 9 wild-type, n = 7 Dp1Tyb) and female (n = 9 wild-type, n = 9 Dp1Tyb) were used. For the endocortical surface, 16-week-old male (n = 10 wild-type, n = 8 Dp1Tyb) and female (n = 11 wild-type, n = 10 Dp1Tyb) were used.

For the static histomorphometry, sections of the distal femur were deplasticsized in acetone and stained for osteoid using the Von Kossa-MacNeal's tetrachrome protocol or osteoclasts using TRAP staining. Osteoclast surface to bone surface (OcS/BS), osteoclast number per 1 mm tissue (Oc#/BS), and osteoid surface/bone surface (OS/BS) were quantified using Bioquant software. Sixteen-week-old male (n = 13 control, n = 10 Dp1Tyb) and female (n = 11 control, n = 12 Dp1Tyb) were used for osteoclast analysis and 16-week-old male (n = 13 control, n = 11 Dp1Tyb) and female (n = 13 control, n = 12 Dp1Tyb) were used for osteoid analysis.

### 2.6 Statistical tests

MicroCT and mechanical testing data were analyzed using a custom Matlab code [45]. For microCT, mechanical testing, and histomorphometry data, normality and homogeneity were assessed for each phenotype and any violations were transformed to achieve normality. To control for the analysis of multiple dependent variables, separate MANOVAs (Wilks' Lambda, IBM SPSS version 26) were performed on the 6- and 16-week-old mice for trabecular (4 parameters), cortical (14 parameters) and mechanical (16 parameters) data. If the MANOVA for a given age and bone type yielded significant main or interactive effects of genotype and/or sex, follow-up 2-way factorial ANOVAs were performed on each individual parameter to identify which of the individual parameters expressed the main (or interactive) effects that were significant in the MANOVA. If the MANOVA yielded no significant effects, no additional follow-up analyses were performed. Effect sizes were obtained from the SPSS calculations of partial eta squared in each ANOVA. For each parameter with a significant interaction, post hoc analyses (Tukey's multiple comparisons test) determined differences between groups.

## 3 Results

### 3.1 Preliminary skeletal analyses

Preliminary analyses on mice at 14 weeks showed an effect of both sex (p < 0.001) and genotype (trisomy or extra gene dosage) on the length from nose to base of tail (p = 0.001) (Dp1Tyb male = 89.84 (SE ± 0.86) mm; control male = 93.12 (0.94) mm; Dp1Tyb female = 85.24 (1.15) mm; control female = 88.56 (0.41) mm; n = 10 for all samples). Male and control mice overall showed a longer body at 14 weeks. The length of the femur was greater in wild-type mice as compared to Dp1Tyb mice at 14 weeks (p < 0.001) (Dp1Tyb male = 14.78 (0.20) mm; control male = 15.72 (0.20) mm; Dp1Tyb female = 14.76 (0.22); control female = 15.40 (0.18); n = 10 for all samples).

### 3.2 Interaction of sex and genotype in Dp1Tyb mice alters BV/TV during skeletal maturation

**Fig. 1:**
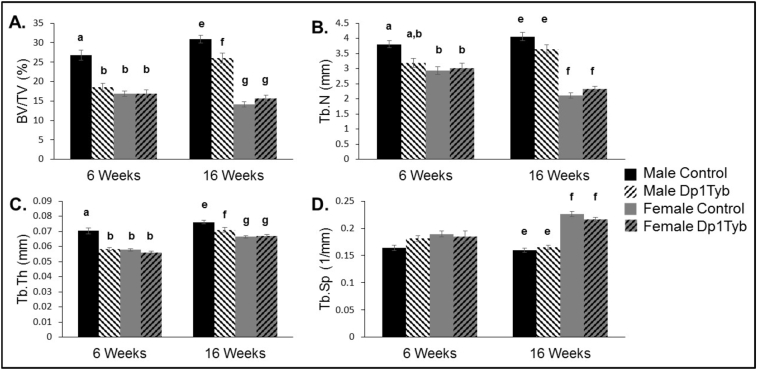
Trabecular measures on Dp1Tyb and littermate control femurs at 6 and 16 weeks (mean ± SEM). MANOVAs on the combined four trabecular parameters, performed separately for the 6- and 16-week data, respectively, indicated significant sex × genotype interactive effects (p = 0.001, p = 0.001), along with significant main effects of sex (p < 0.001, p < 0.001) and genotype (p < 0.001, p = 0.003). The individual parameters are depicted in the four panels: (A) Percent bone volume (BV/TV); 6 weeks: sex × genotype interaction: p = 0.044, [η_p_^2^ = 0.103]; genotypic effect: p = 0.001, [η_p_^2^ = 0.275], and sex effect: p < 0.001, [η_p_^2^ = 0.426]; 16 weeks: sex × genotype interaction: p = 0.001, [η_p_^2^ = 0.164]; genotypic effect: p = 0.069, [η_p_^2^ = 0.056], and sex effect: p < 0.001, η_p_^2^ = 0.785. (B) Trabecular number (Tb.N): 6 weeks: sex × genotype interaction: p < 0.001, [η_p_^2^ = 0.268]; genotypic effect: p = 0.097, [η_p_^2^ = 0.071], and sex effect: p = 0.003, [η_p_^2^ = 0.209]; 16 weeks: sex × genotype interaction: p = 0.009, [η_p_^2^ = 0.112]; genotypic effect: p = 0.345, [η_p_^2^ = 0.015], and sex effect: p < 0.001, η_p_^2^ = 0.779. (C) Trabecular thickness (Tb.Th): 6 weeks: sex × genotype interaction: p < 0.001, [η_p_^2^ = 0.283]; genotypic effect: p < 0.001, [η_p_^2^ = 0.413], and sex effect: p < 0.001, [η_p_^2^ = 0.446]; 16 weeks: sex × genotype interaction: p = 0.015, [η_p_^2^ = 0.097]; genotypic effect: p = 0.021, [η_p_^2^ = 0.089], and sex effect: p < 0.001, [η_p_^2^ = 0.420]. (D) Trabecular separation (Tb.Sp): 6 weeks: sex × genotype interaction: p = 0.174, [η_p_^2^ = 0.048]; genotypic effect: p = 0.431, [η_p_^2^ = 0.016], and sex effect: p = 0.063, [η_p_^2^ = 0.088]; 16 weeks: sex × genotype interaction: p = 0.098, [η_p_^2^ = 0.046]; genotypic effect: p = 0.669, [η_p_^2^ = 0.003], and sex effect: p < 0.001, [η_p_^2^ = 0.736]. Male control (n = 10 6 wk.; n = 13 16 wk); Male Dp1Tyb; n = 10 6 wk.; n = 11 16 wk); Female control (n = 12 6 wk.; n = 22 16 wk); Female Dp1Tyb (n = 10 6 wk.; n = 16 16 wk). Significant differences and interactions for individual parameters are as determined by ANOVA and the p value is followed by partial eta squared [η_p_^2^] as a measure of effect size. Similarities and differences between individual groups are determined from Tukey's multiple comparisons post hoc tests; values with the same superscript letter are not significantly different. Letters a,b,c,d are used for comparisons of 6 week old animals; letters e,f,g,h are used for comparisons of 16 week old animals.

The interaction of sex (male vs. female) and genotype (three copy vs. normal copy number) was important in percent bone volume (bone volume/tissue volume or BV/TV) at both 6 and 16 weeks of age. At 6 weeks of age during longitudinal bone growth, BV/TV was reduced in male Dp1Tyb, female Dp1Tyb and female control animals as compared to male control animals (Fig. 1A). At 16 weeks of age, a time of skeletal maturity, BV/TV continued to be reduced in male Dp1Tyb, female Dp1Tyb and female control animals as compared to male control animals. Additionally, female Dp1Tyb mice had reduced BV/TV as compared to male Dp1Tyb mice (Fig. 1A). BV/TV increased in male mice from 6 to 16 weeks of age (p < 0.001), but female mice exhibited a loss in BV/TV during the same time period (p = 0.025). Similar data were observed for BMD in the trabecular compartment at both 6 and 16 weeks. Taken together, these data indicate that percent bone volume is reduced in Dp1Tyb as compared to wild-type male mice as the bone is actively growing, and approximately when skeletal maturity is achieved. Female mice have reduced formation or bone accrual independent of genotype at 6 weeks. At 16 weeks of age, as the appendicular skeleton reaches skeletal maturity, BV/TV is increased in male mice, but decreased in female mice as compared to 6 weeks of age.

### 3.3 Interaction between sex and genotype affects trabecular microarchitecture in male and female Dp1Tyb and wild-type mice

Measurements of other trabecular skeletal parameters provide insight into the interaction between sex and genotype. Trabecular number (Tb.N) is increased in male wild-type mice compared to female Dp1Tyb and wild-type mice at 6 and 16 weeks (Fig. 1B) and trabecular thickness (Tb.Th) is greater in male wild-type mice than male and female Dp1Tyb and wild-type mice at both 6 and 16 weeks (Fig. 1C). Trabecular separation (Tb.Sp) is greater in female than male mice at 16 weeks (Fig. 1D). In male mice, Tb.N increased (p = 0.013) and Tb.Sp decreased (p = 0.034) from 6 to 16 weeks for both genotypes. Tb.Th showed an age × genotype interaction (p = 0.021) in male mice. When only female mice were analyzed together at 6 and 16 weeks, Tb.N decreased and Tb.Th and Tb.Sp increased (p < 0.001 for all). These data together imply that both male control and Dp1Tyb mice develop trabecular bone between 6 and 16 weeks consistent with a normal growth pattern, however Dp1Tyb mice exhibit structural deficits compared to control mice. Female mice gain Tb.Th and Tb.Sp and lose Tb.N over time, indicating reduced bone modeling or increased resorption across ages.

### 3.4 Cortical bone parameters exhibit differences between sex and genotype in Dp1Tyb and control mice at 6 weeks, and interactive effects at 16 weeks

**Fig. 2:**
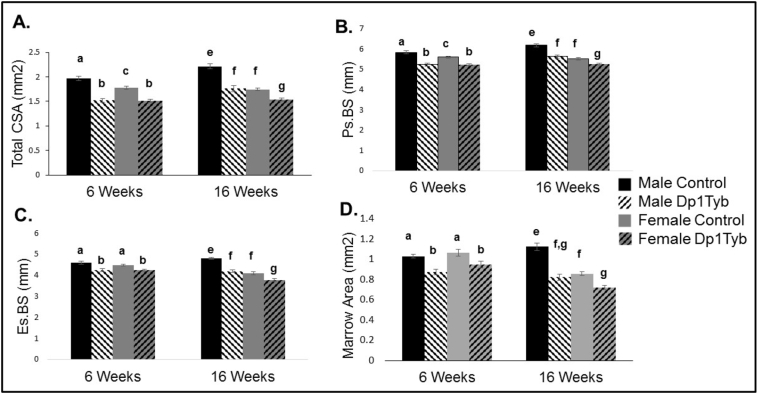
Cortical measures on Dp1Tyb and control femurs at 6 and 16 weeks (mean ± SEM). MANOVAs on the combined 16 cortical parameters, performed separately for the 6- and 16-week cortical data, respectively, indicated significant main effects of sex (p = 0.003, p < 0.01) and genotype (p < 0.001, p < 0.001). The interaction between sex and genotype was significant at 16 weeks p = p < 0.001) but not 6 weeks (p = 0.236). Four of the 16 individual parameters showing significant sex × genotype interactions at 16 weeks are depicted in the four panels: (A) Cortical total cross-sectional area (CSA): 6 weeks: sex × genotype interaction: p = 0.024, [η_p_^2^ = 0.127]; genotypic effect: p < 0.001, [η_p_^2^ = 0.689], and sex effect: p = 0.014, [η_p_^2^ = 0.149]; 16 weeks: sex × genotype interaction: p = 0.001, [η_p_^2^ = 0.177]; genotypic effect: p < 0.001, [η_p_^2^ = 0.613], and sex effect: p < 0.001, [η_p_^2^ = 0.574]. (B) Periosteal bone surface (Ps.BS): 6 weeks: sex × genotype interaction: p = 0.070, [η_p_^2^ = 0.084]; genotypic effect: p < 0.001, [η_p_^2^ = 0.619], and sex effect: p = 0.060, [η_p_^2^ = 0.090]; 16 weeks: sex × genotype interaction: p = 0.007, [η_p_^2^ = 0.118]; genotypic effect: p < 0.001, [η_p_^2^ = 0.590], and sex effect: p < 0.001, [η_p_^2^ = 0.624]. (C) Endocortical bone surface (Es.BS): 6 weeks: sex × genotype interaction: p = 0.343, [η_p_^2^ = 0.024]; genotypic effect: p < 0.001, [η_p_^2^ = 0.396], and sex effect: p = 0.319, [η_p_^2^ = 0.026]; 16 weeks: sex × genotype interaction: p = 0.013, [η_p_^2^ = 0.101]; genotypic effect: p < 0.001, [η_p_^2^ = 0.590], and sex effect: p < 0.001, [η_p_^2^ = 0.635]. (D) Marrow area: 6 weeks: sex × genotype interaction: p = 0.157, [η_p_^2^ = 0.052]; genotypic effect: p < 0.001, [η_p_^2^ = 0.430], and sex effect: p = 0.238, [η_p_^2^ = 0.036]; 16 weeks: sex × genotype interaction: p = 0.002, [η_p_^2^ = 0.157]; genotypic effect: p < 0.001, [η_p_^2^ = 0.607], and sex effect: p < 0.001, [η_p_^2^ = 0.467] in Dp1Tyb and control male and female mice at 6 and 16 weeks. Significant differences and interactions for individual parameters are as determined by ANOVA and the p value is followed by partial eta squared [η_p_^2^] as a measure of effect size. Similarities and differences between individual groups are determined from Tukey's multiple comparisons post hoc tests; values with the same superscript letter are not significantly different. Letters a,b,c,d are used for comparisons of 6 week old animals; letters e,f,g,h are used for comparisons of 16 week old animals.

Six week-old control male and female mice had a greater total cross-sectional area (total CSA) in the cortical bone than Dp1Tyb male and female mice (Fig. 2A). Total CSA increased from 6 to 16 weeks in male (p < 0.001) but not female (p = 0.61) mice. At 16 weeks of age, total CSA is larger in control male than all other mice, male Dp1Tyb as compared to female Dp1Tyb mice, and female control as compared to female Dp1Tyb mice.

The sexual dimorphism and effects of trisomic gene dosage in total CSA were further understood by examining other cortical parameters. At 6 weeks of age, all control as compared Dp1Tyb mice together had a larger marrow area, cortical area, cortical thickness, periosteal bone surface (Ps.BS), endosteal bone surface (Es.BS), Imax, and Imin. Male as compared to female mice overall exhibited greater cortical area, Imax and Imin (Table 1 & Fig. 2). In addition to a reduction in cortical structural parameters, Dp1Tyb mice exhibited lower tissue mineral density (TMD) compared to their wild-type counterparts.

**Table 1:** Cortical architecture and geometry of Dp1Tyb and control male and female mice at 6 and 16 weeks.

	Male control 6 weeks	Male Dp1Tyb 6 weeks	Female control 6 weeks	Female Dp1Tyb 6 weeks	Between subjects effects Interaction	Between subjects effects Genotype	Between subjects effects Sex
	n = 10	n = 10	n = 12	n = 10			
Cortical area (mm^2^)	0.82 (0.03)^a^	0.58 (0.02)^b^	0.71 (0.03)^c^	0.56 (0.02)^b^	p = 0.091 [η_p_^2^ = 0.074]	p < 0.001 [η_p_^2^ = 0.566]	p = 0.026 [η_p_^2^ = 0.124]
Cortical thickness (mm)	0.19 (0.005)^a^	0.15 (0.003)^b^	0.17 (0.008)^a,b^	0.15 (0.007)^b^	p = 0.270 [η_p_^2^ = 0.032]	p < 0.001 [η_p_^2^ = 0.367]	p = 0.139 [η_p_^2^ = 0.057]
Imax (mm^4^)	0.27 (0.01)^a^	0.16 (0.01)^b^	0.22 (0.01)^c^	0.15 (0.01)^b^	p = 0.075 [η_p_^2^ = 0.081]	p < 0.001 [η_p_^2^ = 0.585]	p = 0.019 [η_p_^2^ = 0.137]
Imin (mm^4^)	0.15 (0.01)^a^	0.08 (0.004)^b^	0.12 (0.005)^c^	0.08 (0.003)^b^	p = 0.004 [η_p_^2^ = 0.202]	p < 0.001 [η_p_^2^ = 0.740]	p = 0.001 [η_p_^2^ = 0.253]
TMD (g/cm^3^ HA)	1.05 (0.01)^a^	0.99 (0.01)^b^	1.05 (0.02)^a^	1.01 (0.02)^a,b^	p = 0.655 [η_p_^2^ = 0.005]	p = 0.001 [η_p_^2^ = 0.253]	p = 0.592 [η_p_^2^ = 0.008]


	Male control 16 weeks	Male Dp1Tyb 16 weeks	Female control 16 weeks	Female Dp1Tyb 16 weeks	Between subjects effects Interaction	Between subjects effects Genotype	Between subjects effects Sex
	n = 13	n = 12	n = 20	n = 22			
Cortical area (mm^2^)	1.09 (0.02)^e^	0.92 (0.02)^f^	0.89 (0.01)^f^	0.82 (0.01)^g^	p = 0.005 [η_p_^2^ = 0.130]	p < 0.001 [η_p_^2^ = 0.476]	p < 0.001 [η_p_^2^ = 0.585]
Cortical thickness (mm)	0.24 (0.003)^e^	0.24 (0.004)^e^	0.22 (0.002)^f^	0.22 (0.002)^f^	p = 0.184 [η_p_^2^ = 0.0.30]	p = 0.136 [η_p_^2^ = 0.038]	p < 0.001 [η_p_^2^ = 0.341]
Imax (mm^4^)	0.43 (0.02)^e^	0.29 (0.02)^f^	0.26 (0.01)^f^	0.21 (0.01)^g^	p = 0.001 [η_p_^2^ = 0.189]	p < 0.001 [η_p_^2^ = 0.543]	p < 0.001 [η_p_^2^ = 0.681]
Imin (mm^4^)	0.20 (0.01)^e^	0.12 (0.005)^f,g^	0.13 (0.004)^f^	0.11 (0.004)^g^	p < 0.001 [η_p_^2^ = 0.241]	p < 0.001 [η_p_^2^ = 0.609]	p < 0.001 [η_p_^2^ = 0.452]
TMD (g/cm^3^ HA)	1.14 (0.02)	1.14 (0.03)	1.15 (0.02)	1.17 (0.02)	p = 0.733 [η_p_^2^ = 0.002]	p = 0.889 [η_p_^2^ = 0.000]	p = 0.573 [η_p_^2^ = 0.006]

Values are averages (SEM). Similarities and differences between individual groups as determined from Tukey's multiple comparisons post hoc tests; values with the same superscript letter are not significantly different. Letters a,b,c,d are used for comparisons of 6 week old animals; letters e,f,g,h are used for comparisons of 16 week old animals. Data without letters within a single row are not significantly different from each other. Subject effects are p values and partial eta squared (η_p_^2^) in brackets.

At 16 weeks of age, male control mice had larger total CSA, cortical area, Ps.BS, Es.BS and Imax compared to all other mice, with male mice as a group with higher parameters compared to female mice, and female Dp1Tyb mice having the lowest measurements (Table 1 & Fig. 2). Marrow area and Imin are greater in male control than all other mice, and female control are greater than female Dp1Tyb mice for these same parameters. Female mice as a group had reduced cortical thickness compared to male mice. These data suggest that the smaller total CSA in male Dp1Tyb as compared to control mice at 6 and 16 weeks is due to reduced Ps.BS, Es.BS, cortical thickness and marrow area at both ages. In female mice, total CSA appears unchanged and cortical area and thickness is increased from 6 to 16 weeks with decreases in Es.BS and marrow area.

### 3.5 Mechanical bone parameters exhibit a sexual dimorphism in extrinsic bone properties in Dp1Tyb and control mice

**Fig. 3:**
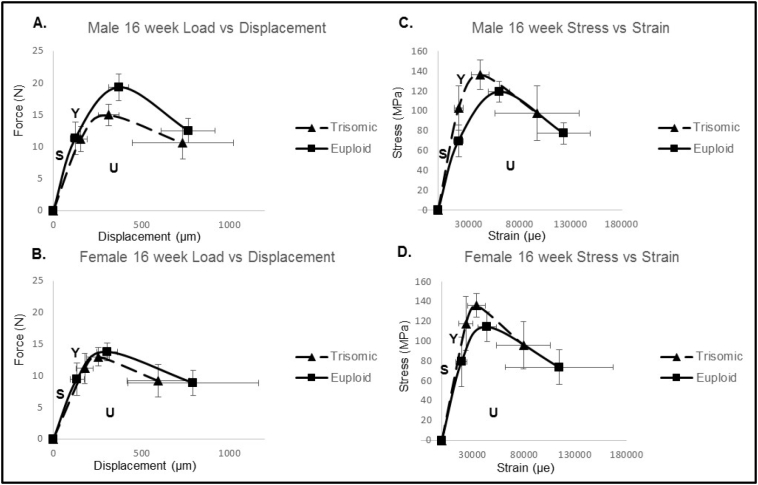
Schematic representation of mechanical testing curves (data represented by mean ± SEM). A and B) Representations of force-displacement curves at the structural level. C and D) Representations of stress-strain curves at the tissue level. S, is related to the stiffness or modulus of the femur. Y, the yield point represents elastic behavior, indicating the bone's resistance to permanent deformation, past this point, U is the plastic region or permanent deformation where the bone has sustained permanent damage.

The interactive effect and main effect of genotype were significant when all mechanical parameters at 6 weeks were analyzed together by MANOVA, and the sex effect was marginal (p = 0.100). Interactive, sex, and genotype effects were all significant at 16 weeks when mechanical parameters were analyzed together by MANOVA. At 6 weeks of age in the mechanical properties of extrinsic bone (properties based on bone mass and geometry), the only sex × genotype interaction was for stiffness, with male euploid mice having greater stiffness than all other mice. Control as compared to Dp1Tyb mice at 6 weeks exhibited higher yield force (bone undergoes permanent deformation), ultimate force (measuring general strength of bone), postyield work (energy absorbed after permanent deformation), and total work (total energy absorbed by the bone during bending). Male as compared to female 6-week-old mice also showed a higher ultimate force. (Table 2 and Fig. 3). At 16 weeks of age, the only sex × genotype interaction for extrinsic bone properties was for ultimate force, with control males greater than all other mice and Dp1Tyb males greater than Dp1Tyb females. Control as compared to Dp1Tyb mice at 16 weeks of age had a higher postyield displacement, stiffness, postyield work, and total work; and Dp1Tyb as compared to control mice displayed higher values of displacement to yield and work to yield. At 16 weeks of age, males as compared to females had higher measurements for stiffness, postyield work, and total work (Table 3 and Fig. 3). Taken together, these data indicate that there was a strong genotypic effect at 6 weeks in extrinsic bone properties but at 16 weeks, sex and especially genotype had non-interactive effects on overall bone properties. At 16 weeks of age, additional gene dosage and female sex were detrimental to whole bone properties.

**Table 2:** Mechanical testing at 6 weeks.

	Male control 6 week	Male Dp1Tyb 6 week	Female control 6 week	Female Dp1Tyb 6 week	Between subjects effects Interaction	Between subjects effects Genotype	Between subjects effects Sex
	n = 10	n = 10	n = 12	n = 10			
Yield force (N)	6.53 (0.68)	5.26 (0.52)	6.92 (1.08)	4.95 (0.54)	p = 0.663 [η_p_^2^ = 0.005]	p = 0.048 [η_p_^2^ = 0.099]	p = 0.958 [η_p_^2^ = 0.000]
Ultimate force (N)	12.90 (0.71)^a^	7.87 (0.34)^b^	10.30 (0.77)^c^	7.51 (0.51)^b^	p = 0.083 [η_p_^2^ = 0.077]	p < 0.001 [η_p_^2^ = 0.503]	p = 0.024 [η_p_^2^ = 0.127]
Displacement to yield (μm)	104.40 (10.71)	139.10 (13.51)	149.08 (26.28)	150.0 (25.78)	p = 0.430 [η_p_^2^ = 0.016]	p = 0.405 [η_p_^2^ = 0.018]	p = 0.197 [η_p_^2^ = 0.043]
Postyield displacement (μm)	481.80 (22.66)	464.70 (38.43)	487.42 (32.24)	664.60 (176.05)	p = 0.279 [η_p_^2^ = 0.031]	p = 0.371 [η_p_^2^ = 0.021]	p = 0.253 [η_p_^2^ = 0.034]
Total displacement (μm)	586.20 (20.94)	603.80 (36.62)	636.50 (26.11)	814.60 (171.38)	p = 0.353 [η_p_^2^ = 0.023]	p = 0.259 [η_p_^2^ = 0.033]	p = 0.135 [η_p_^2^ = 0.058]
Stiffness (N/mm)	76.81 (5.58)^a^	43.28 (2.42)^b^	55.26 (3.36)^b^	45.13 (8.23)^b^	p = 0.032 [η_p_^2^ = 0.116]	p < 0.001 [η_p_^2^ = 0.313]	p = 0.068 [η_p_^2^ = 0.085]
Work to yield (mJ)	0.40 (0.07)	0.44 (0.09)	0.79 (0.26)	0.49 (0.14)	p = 0.332 [η_p_^2^ = 0.025]	p = 0.463 [η_p_^2^ = 0.014]	p = 0.208 [η_p_^2^ = 0.041]
Postyield work (mJ)	5.39 (0.34)^a^	3.25 (0.29)^b^	4.31 (0.37)^a,b^	3.55 (0.47)^b^	p = 0.072 [η_p_^2^ = 0.083]	p < 0.001 [η_p_^2^ = 0.281]	p = 0.306 [η_p_^2^ = 0.028]
Total work (mJ)	5.79 (0.35)^a^	3.69 (0.28)^b^	5.09 (0.51)^a,b^	4.04 (0.46)^b^	p = 0.223 [η_p_^2^ = 0.039]	p = 0.001 [η_p_^2^ = 0.267]	p = 0.693 [η_p_^2^ = 0.004]
Yield stress (MPa)	58.02 (6.09)	76.45 (7.79)	69.84 (6.93)	72.97 (7.39)	p = 0.289 [η_p_^2^ = 0.030]	p = 0.138 [η_p_^2^ = 0.057]	p = 0.561 [η_p_^2^ = 0.009]
Ultimate stress (MPa)	112.41 (2.44)	113.16 (4.09)	108.19 (3.67)	109.74 (3.54)	p = 0.911 [η_p_^2^ = 0.000]	p = 0.746 [η_p_^2^ = 0.003]	p = 0.286 [η_p_^2^ = 0.030]
Strain to yield (μe)	17,021.85 (1695.62)	20,148.02 (1886.03)	23,784.06 (4115.67)	21,617.10 (3690.98)	p = 0.412 [η_p_^2^ = 0.018]	p = 0.881 [η_p_^2^ = 0.001]	p = 0.205 [η_p_^2^ = 0.042]
Total strain (μe)	96,021.77 (3554.68)	87,969.07 (5732.33)	102,403.00 (4508.62)	117,812.69 (24,825.71)	p = 0.355 [η_p_^2^ = 0.023]	p = 0.771 [η_p_^2^ = 0.002]	p = 0.156 [η_p_^2^ = 0.052]
Modulus (GPa)	4.08 (0.18)	4.27 (0.17)	3.71 (0.27)	4.41 (0.59)	p = 0.453 [η_p_^2^ = 0.015]	p = 0.628 [η_p_^2^ = 0.006]	p = 0.751 [η_p_^2^ = 0.003]
Resilience (MPa)	0.58 (0.11)	0.93 (0.18)	1.17 (0.34)	1.08 (0.32)	p = 0.416 [η_p_^2^ = 0.018]	p = 0.627 [η_p_^2^ = 0.006]	p = 0.168 [η_p_^2^ = 0.049]

Values are averages (SEM). Similarities and differences between individual groups as determined from Tukey's multiple comparisons post hoc tests; values with the same superscript letter are not significantly different. Data without letters within a single row are not significantly different from each other. Subject effects are p values and partial eta squared (η_p_^2^) in brackets.

**Table 3:** Mechanical testing at 16 weeks.

	Male control 16 week	Male Dp1Tyb 16 week	Female control 16 week	Female Dp1Tyb 16 week	Between subjects effects Interaction	Between subjects effects Genotype	Between subjects effects Sex
	n = 13	n = 12	n = 20	n = 22			
Yield force (N)	11.30 (0.70)	11.19 (0.61)	9.42 (0.55)	11.14 (0.59)	p = 0.158 [η_p_^2^ = 0.034]	p = 0.211 [η_p_^2^ = 0.027]	p = 0.134 [η_p_^2^ = 0.038]
Ultimate force (N)	19.34 (0.58)^e^	14.98 (0.52)^f^	13.75 (0.30)^f,g^	12.98 (0.37)^g^	p < 0.001 [η_p_^2^ = 0.231]	p < 0.001 [η_p_^2^ = 0.379]	p < 0.001 [η_p_^2^ = 0.572]
Displacement to yield (μm)	123.46 (7.69)^e^	154.82 (11.22)^e,f^	133.18 (8.41)^e^	177.06 (12.29)^f^	p = 0.552 [η_p_^2^ = 0.006]	p = 0.001 [η_p_^2^ = 0.182]	p = 0.132 [η_p_^2^ = 0.039]
Postyield displacement (μm)	641.54 (43.80)	579.64 (93.59)	660.32 (79.03)	416.94 (50.45)	p = 0.233 [η_p_^2^ = 0.024]	p = 0.047 [η_p_^2^ = 0.066]	p = 0.343 [η_p_^2^ = 0.016]
Total displacement (μm)	765.00 (42.51)	734.45 (86.39)	793.50 (79.51)	594.00 (44.07)	p = 0.252 [η_p_^2^ = 0.023]	p = 0.121 [η_p_^2^ = 0.041]	p = 0.447 [η_p_^2^ = 0.010]
Stiffness (N/mm)	102.79 (4.79)^e^	82.66 (2.88)^f^	82.45 (3.92)^f^	70.22 (2.99)^f^	p = 0.333 [η_p_^2^ = 0.016]	p < 0.001 [η_p_^2^ = 0.217]	p < 0.001 [η_p_^2^ = 0.221]
Work to yield (mJ)	0.82 (0.10)^e,f^	0.99 (0.15)^e,f^	0.74 (0.08)^e^	1.13 (0.13)^f^	p = 0.335 [η_p_^2^ = 0.016]	p = 0.021 [η_p_^2^ = 0.088]	p = 0.813 [η_p_^2^ = 0.001]
Postyield work (mJ)	10.09 (0.64)^e^	7.20 (1.12)^e,f,g^	7.17 (0.71)^f^	4.42 (0.53)^g^	p = 0.925 [η_p_^2^ = 0.000]	p = 0.001 [η_p_^2^ = 0.186]	p = 0.001 [η_p_^2^ = 0.190]
Total work (mJ)	10.92 (0.64)^e^	8.19 (1.04)^e,f^	7.91 (0.71)^f^	5.55 (0.46)^f^	p = 0.802 [η_p_^2^ = 0.001]	p = 0.001 [η_p_^2^ = 0.168]	p < 0.001 [η_p_^2^ = 0.200]
Yield stress (MPa)	69.71 (4.43)^e^	102.86 (6.69)^f^	79.16 (5.31)^e^	117.78 (6.83)^f^	p = 0.790 [η_p_^2^ = 0.001]	p < 0.001 [η_p_^2^ = 0.367]	p = 0.054 [η_p_^2^ = 0.063]
Ultimate stress (MPa)	119.20 (2.85)^e^	136.40 (4.51)^f^	114.28 (3.17)^e^	135.84 (2.97)^g^	p = 0.660 [η_p_^2^ = 0.003]	p < 0.001 [η_p_^2^ = 0.346]	p = 0.436 [η_p_^2^ = 0.010]
Strain to yield (μe)	19,855.75 (1323.57)^e^	20,087.80 (1347.45)	19,147.71 (1162.29)	23,488.38 (1666.11)	p = 0.536 [η_p_^2^ = 0.007]	p = 0.120 [η_p_^2^ = 0.041]	p = 0.356 [η_p_^2^ = 0.015]
Total strain (μe)	122,902.16 (7310.71)^e^	97,030.47 (12,452.32)^e,f^	114,524.62 (11,225.61)^e,f^	79,660.87 (6600.21)^f^	p = 0.672 [η_p_^2^ = 0.003]	p = 0.006 [η_p_^2^ = 0.124]	p = 0.228 [η_p_^2^ = 0.025]
Modulus (GPa)	3.95 (0.18)^e^	5.79 (0.22)^f^	4.68 (0.18)^g^	5.55 (0.21)^f^	p = 0.022 [η_p_^2^ = 0.088]	p < 0.001 [η_p_^2^ = 0.428]	p = 0.246 [η_p_^2^ = 0.023]
Resilience (MPa)	0.83 (0.12)^e^	1.18 (0.17)^e,f^	0.90 (0.11)^e^	1.59 (0.18)^f^	p = 0.267 [η_p_^2^ = 0.021]	p = 0.001 [η_p_^2^ = 0.172]	p = 0.110 [η_p_^2^ = 0.044]

Values are averages (SEM). Similarities and differences between individual groups as determined from Tukey's multiple comparisons post hoc tests; values with the same superscript letter are not significantly different. Data without letters within a single row are not significantly different from each other. Subject effects are p values and partial eta squared (η_p_^2^) in brackets.

### 3.6 Dp1Tyb and female mice appear to have improved intrinsic bone properties

At 6 weeks of age there were no effects in the intrinsic properties (material properties independent of size and shape) of bone in Dp1Tyb as compared to control or male as compared to female mice. At 16 weeks, control males had a lower modulus (stiffness of material) than all other mice (sex × genotype interaction), and both female and male Dp1Tyb mice had a higher modulus than their control littermates (Table 3 and Fig. 3). Also at 16 weeks, bone from Dp1Tyb as compared to control mice displayed a higher yield stress (normalized force to size and shape of bone), ultimate stress, and resilience. Control as compared to Dp1Tyb mice had a higher total strain. Female mice had marginally increased yield stress as compared to male mice. Taken together, these data indicate that at 16 weeks, Dp1Tyb and female mice seem to have improved material bone properties as compared to control and male mice, especially in the elastic region before the bone becomes permanently deformed.

### 3.7 Histomorphometric analysis of cellular properties show sex effects in Dp1Tyb and control mice

**Fig. 4:**
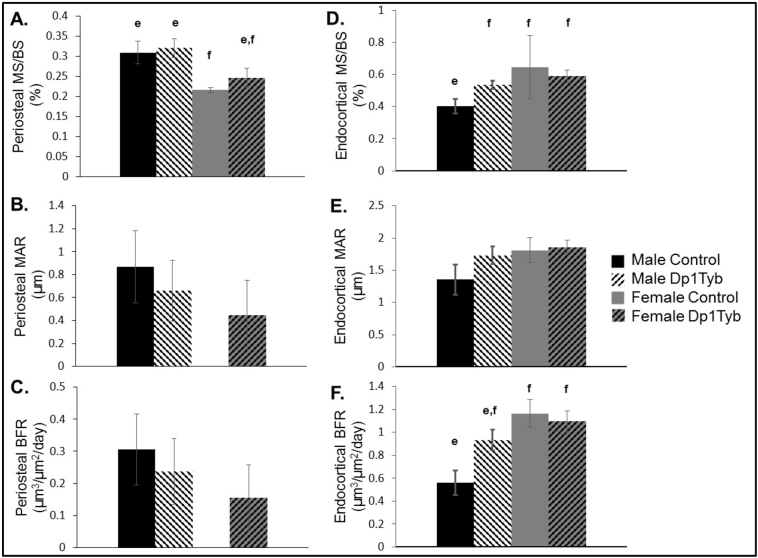
Dynamic bone labeling measures of the cortical region of Dp1Tyb and control femurs at 16 weeks of age (data represented by mean ± SEM). (A) Periosteal mineralizing surface/bone surface (MS/BS), (B) Periosteal mineral apposition rate (MAR), (C) Periosteal bone formation rate (BFR), (D) Endocortical MS/BS, (E) Endocortical MAR, (F) Endocortical BFR. In (B) and (C), there were no measurable effects in female control MAR and BFR (9 samples in each group). Similarities and differences between individual groups are determined from Tukey's multiple comparisons post hoc tests; values with the same superscript letter are not significantly different. Letters a,b,c,d are used for comparisons of 6 week old animals; letters e,f,g,h are used for comparisons of 16 week old animals.

To understand more about the cellular properties of the femur at 16 weeks when sex and genotypic dimorphisms in intrinsic and extrinsic properties are observed, we analyzed dynamic properties in both cortical (femur midshaft) and trabecular (distal femur) bone. In addition, but not related to mechanical assessment, we analyzed static properties in the trabecular bone of 16-week-old male and female Dp1Tyb and control littermate mice. In cortical bone at the femoral midshaft on the periosteal surface, male as compared to female mice showed increased MS/BS (p < 0.001, percentage of bone undergoing active formation, and an estimate of osteoblast activity), BFR (p = 0.04, measure of the total rate of new bone formation on the surface being mineralized) and MAR (p = 0.04, osteoblast vigor, the rate at which osteoblasts lay down new bone matrix or osteoid) (Fig. 4A–C). On the endocortical surface, male control as compared to all other mice had a smaller MS/BS (p = 0.008) (sex × genotype interaction). Male control as compared to all female mice had a smaller BFR (p = 0.04) (sex × genotype interaction) (Fig. 4D–F). Taken together, these data support the notion that male mice are more active in forming bone on the periosteal surface while female and trisomic mice are more active in forming bone on the endocortical surface at 16 weeks of age.

**Fig. 5:**
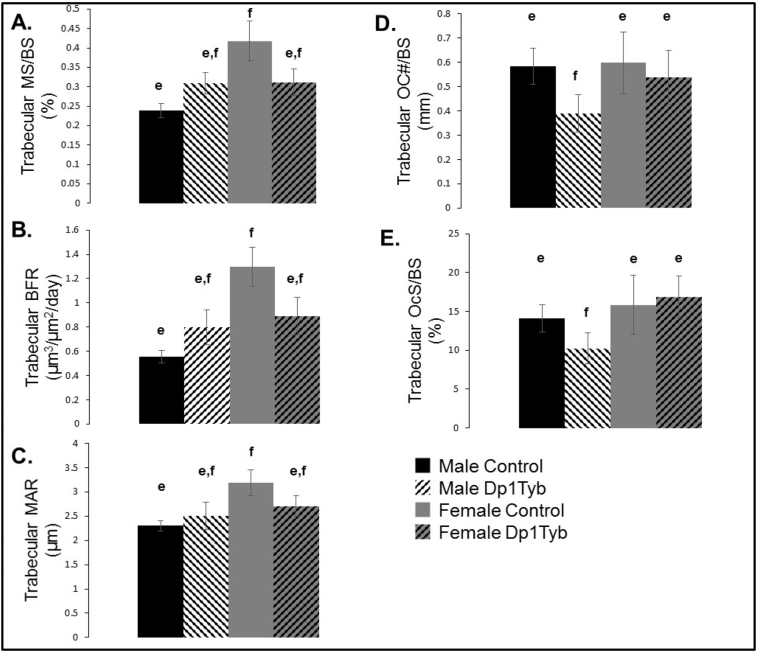
Dynamic bone labeling quantification of the trabecular region of Dp1Tyb and control femurs at 16 weeks of age (data represented by mean ± SEM). (A) Trabecular mineralizing surface/bone surface (MS/BS), (B) Trabecular mineral apposition rate (BFR), (C) Trabecular bone formation rate (MAR), (D) Osteoclast number/bone surface (OC#/BS), (E) Osteoclast surface/bone surface. Similarities and differences between individual groups are determined from Tukey's multiple comparisons post hoc tests; values with the same superscript letter are not significantly different. Letters a,b,c,d are used for comparisons of 6 week old animals; letters e,f,g,h are used for comparisons of 16 week old animals.

In the trabecular bone, there was an increase in female control as compared to male control bone in MS/BS (p = 0.02) and BFR (p = 0.02) (sex × genotype interaction) (Fig. 5A–C). Bone from female as compared to male mice exhibited a higher trabecular MAR (p = 0.02) at 16 weeks of age (Fig. 5C). Osteoid surface per bone surface (OS/BS) (p = 0.04) and OS/BS percentage (p < 0.001) were also greater in trabecular bone of female as compared to male mice. Male Dp1Tyb mice had a lower osteoclast number on the bone surface (Oc#/BS) as compared to all other mice at 16 weeks of age (sex × genotype interaction, p = 0.02) (Fig. 5D) as measured by Tartrate-resistant acid phosphatase (TRAP) labeled multinucleated osteoclasts. Additionally, the percentage of bone surface covered by osteoclasts (OcS/BS) was significantly reduced in male as compared to female (p = 0.004) and control as compared to Dp1Tyb (p = 0.0001) mice (Fig. 5D).

When comparing only mice of the same sex, we observed a marginally increased trabecular osteoblast activity and vigor in 16-week-old male Dp1Tyb male as compared to control mice (MS/BS: p = 0.04, and BFR p = 0.10, 2 tailed *t-*test). In 16-week-old female mice, there was a suggestive decrease in Dp1Tyb as compared to control mice in trabecular MS/BS and BFR (p = 0.11 and p = 0.09, 2 tailed *t-*test). There was reduced trabecular bone osteoclast surface and number in the distal femur of male Dp1Tyb as compared to control littermates at 16 weeks of age (p < 0.001, 1 tailed *t-*test). In female mice at 16 weeks there was a slight reduction in percent osteoclast number in Dp1Tyb as compared to control animals (p = 0.06, 2 tailed *t-*test) and no difference in osteoclast surface between Dp1Tyb and control mice in trabecular bone. No difference was found between male Dp1Tyb and control mice in periosteal cortical MS/BS, MAR, or BFR; there was an increase in Dp1Tyb endosteal MS/BS (p = 0.02, 2 tailed *t-*test) but no differences in endosteal MAR or BFR. There were no significant differences in female periosteal or cortical MS/BS, MAR or BFR. Taken together, these data suggest that in the distal femur at 16 weeks of age, control female mice have the highest osteoblast activity, and Dp1Tyb male mice have the lowest osteoclast activity.

## 4 Discussion

The novel examination of male and female mice in the Dp1Tyb mouse model of DS during typical times of bone accrual and skeletal maturity revealed the effect of both the sex of the mouse and the presence/absence of three copies of dosage sensitive genes on skeletal phenotypes associated with DS. Here we report the first evidence of sexual dimorphism in skeletal deficits of DS model mice that parallels differences between skeletal deficits in humans with DS of different sexes. The non-invasive quantification of bone deficits in humans with DS gives insight into some parameters, including BMD, but does not provide information on the geometry or composition of bone. BMD is a metric used to calculate risk factor for fracture, however, many studies do not include geometry, structural and strength indices that also affect fracture incidence [37,48]. Until recently, studies of bone in individuals with DS have often been done using small sample sizes that may have not been able to detect important changes in bone between males and females with DS during various developmental time points. Using mouse models of DS allows for the quantification of structural, mechanical and cellular properties during different times of bone development. The data obtained show clear age, sexual, and genotypic differences in several measures that recapitulate many phenotypes observed in individuals with DS, especially at formative developmental stages.

At 6 weeks of age, trabecular parameters including BV/TV, Tb.Th, Tb.N and Tb.Sp are adversely affected in both Dp1Tyb and wild-type littermate female mice and are at similar levels as Dp1Tyb male mice. Although females have reduced trabecular measures compared to male wild-type mice at 6 weeks of age, trisomy does not further reduce those measures. These data suggest a protective effect of female sex against trisomic defects during longitudinal bone growth. As mice reach the age of skeletal maturity at 16 weeks, BV/TV of female mice has not increased; furthermore, Tb.N has decreased and Tb.Th and Tb.Sp have increased in both Dp1Tyb and wild-type mice. In female mice, the reduction in Tb.N and increase in Tb.Sp may have negatively influenced connectivity to the point that even with increased Tb.Th at 16 weeks, skeletal measurements are still adversely affected. Taken together, these results show that trisomy has little effect on the trabecular microarchitecture of female mice but could contribute to a decrease in bone formation or an increase in resorption of trabeculae.

At 6 and 16 weeks, Dp1Tyb male and female mice generally have deficits in cortical geometry as compared to their wild-type counterparts. The total CSA increased in male but not female mice from 6 to 16 weeks of age. Measures of cortical bone in male and female Dp1Tyb mice are similar at 6 weeks. Female mice as a group do not have increases of total CSA across ages, but exhibit decreases in marrow area and an increase in cortical area. Dynamic histomorphometry of the femoral midshaft suggests this increase is largely due to increased activity of osteoblasts on the endocortical surface in female mice. Changes in cortical geometry for control and Dp1Tyb mice in the cortical bone follows a normal pattern of skeletal growth, with three copies of genes enhancing deficits in female mice.

### 4.1 Similarities between humans with DS and DS mouse models

It is hypothesized that the osteoporotic phenotype found in individuals with DS is highly influenced by limited bone mineral accretion and reduced peak bone mass attainment during adolescence [11,30]. In humans the age of total body peak bone mass has been estimated to be about 18.8 years in females and 20.5 years in males [49]. Individuals with DS attain peak bone mass earlier and at lower levels than normal individuals [11]. Women with DS (as well as normal women) have bone mineral accrual later than men, but experience rapid bone loss after the age of 40 [30]. Men with DS have a gradual loss of bone after early adulthood, similar to men without DS, but the rate of loss is increased in men with DS [14,30].

Information from the analyses described herein concentrated on skeletal properties during bone accrual (6 weeks) until the estimated age of skeletal maturity in mice (16 weeks) [50]. There are few studies that have examined DS skeletal deficits in humans during adolescence and the time of peak bone accrual, and the results from Dp1Tyb male and female mice may help explain previous reports. The lower BMD seen in adolescent males and females with DS analyzed together as compared to normal individuals [25,26] compares to the genotype effect causing low BV/TV (and BMD) in 6-week Dp1Tyb mice. A study of adolescents with DS that found no differences in BMD between males and females [27] correlates to the similar BV/TV levels in 6 week old male and female Dp1Tyb mice. At an age corresponding to skeletal maturity in individuals with DS, both males and females had a lower BMD [1], with DS females having a lower BMD than DS males in their limbs [2]; both male and female Dp1Tyb mice had significantly affected bone structure at 16 weeks, and trabecular and cortical values were significantly lower in females than males at 16 weeks. The data presented herein also demonstrate that male Dp1Tyb mice exhibit osteopenic phenotypes earlier than their control counterparts, similar to what has been observed in individuals with DS [14,28] and that osteoporotic phenotypes affect males as well as females in early adult stages [30].

### 4.2 Comparison of Dp1Tyb and other DS model mice

Bone deficits associated with DS were first identified in Ts65Dn male mice [36]. A comparison between these and previously published results reveals similar direction and magnitude in trabecular, cortical and mechanical deficits in mice that are at dosage imbalance for genes homologous to Hsa21 [36,39]. Previous studies of 6, 12, 16 week, and 24 month old male mice found no significant differences in trabecular thickness (Tb.Th) between Ts65Dn and euploid mice, though these values were near a p < 0.05 significance level [36,37]. Tb.Th was significantly different between Ts65Dn and control mice at 6 weeks of age and was corrected in Ts65Dn,*Dyrk1a*+/- mice in a subsequent study [39]. The differences in Tb.Th between Dp1Tyb and wild-type mice seem to confirm the dissimilarities in the later study. The data presented confirm that the Dp1Tyb DS mouse model is an effective model for skeletal deficits in male and female DS model mice.

Male and female Ts1Rhr mice, containing 33 triplicated genes, at 3 and 16 weeks have been examined for bone deficits associated with DS [42]. At 3 weeks (a developmental age prior to where we identified differences in BV/TV at 6 weeks in Dp1Tyb and control littermate female mice), BMD was not statistically different between male or female Ts1Rhr or control mice. It may be that BMD is not significant between Ts1Rhr and control male mice at this pre-pubertal age. At 16 weeks, in Ts1Rhr and littermate control mice, areal BMD was not significantly different in the femur, but was decreased in the tibia and increased in the spine. Our analyses of femoral bone in Dp1Tyb and littermate mice found significantly different trabecular BMD between both male and female mice at 16 weeks of age. Further studies, including microCT, were not performed on the bones of Ts1Rhr and control littermate mice, and given the analyses included herein, we hypothesize that other structural and mechanical bone abnormalities exist between Ts1Rhr and control mice. Conversely, the reduction in genes at dosage imbalance in Ts1Rhr as compared to Dp1Tyb mice could dilute any potential effect from triplicated genes.

Differences between skeletal abnormalities could also be due to genetic background, number and which genes are at dosage imbalance, and origin of the change in genetic dosage. Differences in skeletal phenotypes could be due to differences in triplicated gene content or genetic background of Ts65Dn (104 genes, ~50% B6 and ~50% C3H) and Dp1Tyb (148 genes, 100% B6) and Ts1Rhr (33 genes, 100% B6) mice. Differences in trisomy may also contribute to skeletal differences between mouse models. Ts65Dn has a freely segregating extra chromosome with the telomeric end of Mmu16 attached to the centromeric end of Mmu17. This includes ~35 protein-coding genes at dosage imbalance from Mmu17 that are not homologous to Hsa21 [32,40,41]. Both Dp1Tyb and Ts1Rhr mice have a duplication of parts of the distal end of Mmu16 and do not have a freely segregating trisomic chromosome. All of these factors may lead to differences between the DS mouse models that have been used to detect skeletal abnormalities associated with DS. We note that as the gene content of Dp1Tyb mice is known, it may now be possible to look for the causal genes for bone defects, i.e. those sequences on Hsa21 that have an effect when present in three copies.

### 4.3 Mechanical properties of Dp1Tyb mice

Bone is hierarchical and alterations in bone composition, architecture, and cellular activity can increase or decrease bone strength. Our results suggest changes in trabecular microarchitecture and cortical geometry also affected mechanical properties. At 6 weeks of age, most of the significant differences between Dp1Tyb and control mice were found in whole bone properties. In general, control and male mice at 6 and 16 weeks had stronger bones or greater resistance to bending and catastrophic failure compared to their Dp1Tyb counterparts. Genetic dosage imbalance could be sufficient to cause alterations in the developing skeleton of male mice resulting in reduced strength of femur.

At 16 weeks enhanced effects on bone tissue, independent of bone size and shape, were observed. Female as compared to male mice displayed an increase in yield stress but had marginally lower toughness. These increases in material properties of bone in Dp1Tyb mice may indicate brittle bones that are more prone to fracture. Dp1Tyb mice showed an increase in yield properties in both the load-displacement and stress-strain curves. Increases in yield properties are related to bone mineral content and post-yield properties associated with collagen. Dp1Tyb mice had lower toughness, post yield work and total work, suggesting they absorbed less energy prior to fracture and possibly fracture sooner than control animals.

### 4.4 Cellular deficits in mouse models of DS

Quantification of cellular composition in trabecular and cortical bone in 6-week-old male Ts65Dn as compared to control mice showed reduced osteoblast number and/or activity coupled with increased osteoclast activity caused the DS like skeletal deficits at that age [36,39]. A low bone turnover hypothesis was proposed for the deficits skeletal deficits seen in 12-week-old male Ts65Dn mice because of decreased osteoclast and osteoblast activity found on proximal tibia and femur as compared to euploid mice at this age [37]. In 16-week-old male mice, we observed increased osteoblast activity and reduced osteoclast number and activity in the trabecular compartment of Dp1Tyb as compared to control mice. In female mice of the same age, there was a decrease in Dp1Tyb osteoblast activity. These results indicate that osteoblast and osteoclast activity was different in 16-week-old male Dp1Tyb mice than previously reported osteoblast and osteoclast activity in Ts65Dn mice at 6 and 12 weeks. Only a slight increase in osteoblast activity on the periosteal surface of male Dp1Tyb cortical bone was observed. Taken together, these data suggest that sex, gene dosage, bone location and age are all important in the cellular components Dp1Tyb as compared to control mice. Furthermore, the cellular mechanisms leading to bone deficits associated with DS may be distinct at different points in development.

These data demonstrate fundamental differences in skeletal development between Dp1Tyb and wild-type mice. For the first time, they also indicate clear sexual dimorphisms in the development of DS mouse model bone and illustrate time-dependent nature of developmental differences in the DS-associated skeletal deficits. Similarities between the abnormalities in humans with DS and Dp1Tyb mice suggest that skeletal defects result from conserved mechanisms. Sexual dimorphisms, gene dosage, age and bone compartments are all influential in causing alterations in developmental properties that lead to the unique skeletal differences caused by trisomy. These results will help to better understand how trisomy affects individuals with DS and determine which measures may prevent adverse skeletal phenotypes. Additional research is needed to determine when sex-specific skeletal abnormalities develop, how interactions between trisomy and sex influence specific skeletal alterations, and the influence of sex and trisomy on the cellular mechanisms related to these changes in bone development.

## CRediT authorship contribution statement

**Jared R. Thomas:**Methodology, Investigation, Formal analysis, Data curation, Writing - original draft, Project administration.**Jonathan LaCombe:**Methodology, Investigation, Formal analysis, Data curation, Writing - original draft, Project administration.**Rachel Long:**Investigation, Formal analysis, Data curation.**Eva Lana-Elola:**Methodology, Investigation.**Sheona Watson-Scales:**Methodology, Investigation.**Joseph M. Wallace:**Methodology, Formal analysis, Data curation, Writing - original draft.**Elizabeth M.C. Fisher:**Methodology, Investigation.**Victor L.J. Tybulewicz:**Methodology, Investigation.**Randall J. Roper:**Methodology, Investigation, Formal analysis, Data curation, Writing - original draft, Project administration.

## Declaration of competing interest

The authors declare no conflict of interests.
